# Variplex™ test system fails to reliably detect SARS-CoV-2 directly from respiratory samples without RNA extraction

**DOI:** 10.1007/s10096-020-03983-9

**Published:** 2020-07-17

**Authors:** Florian Eckel, Franziska Küsters, Bernhard Drossel, Markus Konert, Hans Mattes, Stefan Schopf

**Affiliations:** 1 Internal Medicine II, Klinikum rechts der Isar, Technische Universität München, Munich, Germany; 2 Medical Department, RoMed Klink Bad Aibling, Harthauser Str. 16, 83043 Bad Aibling, Germany; 3 Hospital Laboratory, RoMed Klink Bad Aibling, Harthauser Str. 16, 83043 Bad Aibling, Germany; 4 Department of Anesthesia, RoMed Klink Bad Aibling, Harthauser Str. 16, 83043 Bad Aibling, Germany; 5 Hospital Hygiene, RoMed Klinikum Rosenheim, Pettenkoferstr. 10, 83022 Rosenheim, Germany; 6 Surgical Department, RoMed Klink Bad Aibling, Harthauser Str. 16, 83043 Bad Aibling, Germany

**Keywords:** Loop-mediated isothermal amplification, COVID-19 diagnostic testing, SARS-CoV-2, Infectious disease outbreaks, Specificity and sensitivity, False-negative reactions

## Abstract

Diagnosis of COVID is performed by PCR methods, but their capacity is limited by the requirement of high-level facilities and instruments. The loop-mediated isothermal amplification (LAMP) method has been utilized for the detection of isolated virus-specific RNA. Preliminary data suggest the possibility of isothermal amplification directly from respiratory samples without RNA extraction. All patients admitted to our hospital were screened for SARS-CoV-2 by routine. Respiratory samples were tested by variplex system based on LAMP method directly without RNA extraction and by PCR. Primary endpoint was the false-negative rate of variplex test compared with PCR as gold standard. In 109 patients variplex test and PCR assay were performed simultaneously. Median age was 80 years and male/female ratio was 40/60%. The prevalence of PCR-confirmed COVID diagnosis was 43.1%. Variplex test was positive in 13.8%. False-negative rate of variplex test compared with PCR was 83.0%. The potential of LAMP technology using isolated RNA has been demonstrated impressively by others, and excellent sensitivity and specificity of detecting SARS-CoV-2 has been reported. However, without RNA extraction, the variplex test system failed to reliably detect SARS-CoV-2 directly in respiratory samples.

## Introduction

Since December 2019, an emerging infectious disease (COVID-19), caused by the novel coronavirus SARS-CoV-2, has emerged in Wuhan, China [1, 2]. As of 1 April 2020, it has caused 876,898 infections in 203 countries, including 43,477 deaths demonstrating the strong human-to-human transmission capacity of SARS-CoV-2. Initially screening focused on patients with foreign travel or contacts with known cases. Both of these foci no longer reflect the current status of the pandemic [3]. The majority of cases have mild or asymptomatic course [4], and symptoms of the COVID-19 infection are highly nonspecific, including respiratory symptoms, fever, cough, dyspnea, and viral pneumonia [5]. Thus, diagnostic tests specific to this infection are urgently required to confirm suspected cases, screen patients, and conduct virus surveillance. In this scenario, a point-of-care device, i.e., a rapid, robust, and cost-efficient device, is crucial and urgently needed for the detection of COVID-19 [6, 7].

At present, the identification of SARS-CoV-2 requires routine and confirmatory diagnosis through real-time polymerase chain reaction (RT-PCR). In recent years, the loop-mediated isothermal amplification (LAMP) method that includes an exponential amplification of specific nucleic acid sequences at a constant temperature, has been widely utilized for the rapid detection of virus-specific genes [8]. The specificity and sensitivity of this method are generally comparable with those of the conventional RT-PCR [9]. The LAMP assays merged with reverse transcription steps have been developed for the detection of RNA viruses, including SARS-CoV-2 [10].

In March 2020, some patients were admitted with suspicion of COVID-19 to our small general hospital in Bavaria, Germany. Patients were treated at one normal care unit of the medical department as well as our interdisciplinary intensive care unit under strict hygiene standards. At that time, delay of RT-PCR, performed by an external institute, for SARS-CoV-2 test results, was 3 days (median, IQR 3–5 days). When a nosocomial SARS-COV-2 outbreak at a normal care unit of the department of trauma surgery and geriatrics was detected, there was urgent need for simple, rapid, and reliable detection of SARS-CoV-2 in our own laboratory.

We decided to use the fast and cheap variplex SARS-CoV-2 test system, which is a ready to use isothermal amplification system for both DNA and RNA using LAMP technology.

As of 1 April 2020, the variplex test system has not received in vitro diagnostic (IVD) certificate. In the meantime it received IVD certificate restricted to the use of isolated RNA. RNA extraction is the first step of any RNA virus testing, such as RT-PCR and isothermal amplifications systems. Commercially available extraction kits make this step more easy; however, RNA extraction may be the limiting step in smaller laboratories in general and may not be available due to the scarcity during pandemic in particular. Therefore, we performed a few variplex tests without RNA extraction directly from respiratory samples. Among this few cases were true positive as well as true negative results. Based on these preliminary promising results and driven by the pressure of the pandemic and a nosocomial SARS-CoV-2 outbreak in our small general hospital, we decided to skip RNA extraction by routine. Here we report on the retrospective analysis of the variplex test system without RNA extraction compared with conventional RT-PCR in all patients admitted to our general hospital in April 2020.

## Materials and methods

This retrospective chart review study involving human participants was in accordance with the ethical standards of the institutional and national research committee and with the 1964 Helsinki Declaration and its later amendments or comparable ethical standards. The Ethics Committee of the Ludwig-Maximilians-University, Munich, Germany, approved this study (approval number 20–432).

During the SARS-CoV-2 pandemic, patients admitted to our hospital were screened for SARS-CoV-2 by routine. Two separate respiratory samples (oropharyngeal or nasopharyngeal swabs) were taken by flexible standard swabs with rayon flocking (MASTASWAB, Mast Group Ltd., Reinfeld, Germany). Samples were sent as dry swabs within 1 h for variplex test in our hospital laboratory and within 24 h for RT-PCR assay to an external laboratory, namely, Medizinisches Labor Rosenheim MVZ GbR, Rosenheim, Germany, member of Limbach Gruppe SE, Heidelberg, Germany.

## LAMP reaction

The variplex SARS-CoV-2 test system (Amplex Diagnostics, Gars-Bahnhof, Germany) was used along with Genie II Mk2 instrument (OptiGene Limited, Horsham, UK). This ready to use test system is based on the loop-mediated isothermal amplification (LAMP) method. Dry swabs (without medium) were dipped, swirled, and squeezed in sputum liquifying solution (SLsolution, Copan Italia, Brescia, Italy) according to the manufacturers advice (preliminary instructions). Variplex test was performed immediately without RNA extraction according to standard procedure provided by Amplex Diagnostics.

A 75 μL (100 μL since 15 April) of the sample in SLSolution was suspended in 500 μL HYPLEX LPTV buffer. A 25 μL reaction mixture for the sample (15 μL Master Mix, 2 μL primer “SARS-CoV-2”, 8 μL LPTV suspension), inhibition control (15 μL Master Mix, 2 μL primer “inhibition control”, 8 μL LPTV suspension), and lysis control (15 μL Master Mix, 10 μL LPTV suspension) was mixed homogeneously. LAMP and fluorescence signal measurements were performed using Genie II thermocycler at 66 °C for 35 min.

## Inclusion and exclusion criteria

Records of all patients admitted to our general hospital from 1 April to 30 April 2020 were analyzed for SARS-CoV-2 routine screening results. Patients were excluded only, if data were incomplete and did not allow comparison of variplex test with RT-PCR-confirmed COVID-19 diagnosis.

## Endpoints

Primary endpoint was the false-negative rate (1-sensitivity) of variplex test compared with PCR assay as gold standard. Secondary endpoints were positive rates of variplex test and RT-PCR assay, sensitivity, specificity, positive and negative predictive value, and accuracy of variplex test compared with RT-PCR assay.

## Statistics

According to the retrospective nature of this study, sample size calculation was not performed. Results are given in numbers and percent, median, and interquartile range (IQR). Exactly 95% confidence interval was calculated by the method of Clopper and Pearson.

## Results

From 1 April to 30 April 2020, 173 patients were admitted to our hospital. In 109 patients included in this study, variplex test and RT-PCR assay were performed simultaneously. Patient characteristics and routine laboratory values on admission were listed in Table 1 and Table 2, respectively.

**Table 1:** Patient characteristics (*n* = 109). Data are median (IQR) or number (%)

Age, years	80 (70–85)
Male/female	44/65 (40.4%/59.6%)
Duration of hospital stay, days	4 (2–7)
Survivors/non-survivors	94/15 (86.2%/13.8%)
Department	
Internal Medicine	84 (77.1%)
Surgery	22 (20.2%)
Otorhinolarynology, head and neck surgery	3 (2.8%)

**Table 2:** Routine laboratory values on admission (*n* = 109)

Laboratory value	Normal value	Median	IQR
White-cell count, /nL	4.3–10.0	7.5	5.5–10.4
Lymphocyte count, /nL	1.30–3.4	0.95	0.69–1.44
Platelet count, /nL	150–350	227	165–276
LDH, U/L	< 248	270	186–355
CRP, mg/dL	< 0.30	3.57	0.78–10.04
High-sensitivity cardiac troponin I, pg/mL	< 60.4	21.8	9.6–45.3
D-dimer, mg/L	< 0.50	1.32	0.76–2.82

LDH = lactate dehydrogenase, CRP = C-reactive protein, IQR = interquartile range

RT-PCR was positive in 47 of 109 patients, resulting in a prevalence of RT-PCR-confirmed COVID-19 diagnosis of 43.1%. Variplex test was positive in 15 of 109 patients (13.8%), thereof positive in 8 of 47 RT-PCR positive patients, resulting in a sensitivity of 17.0% compared with RT-PCR as gold standard. False-negative rate of variplex test was 83.0% (39/47, 69.2% – 92.4%). Sensitivity, specificity, positive and negative predictive value, and accuracy of variplex test compared with RT-PCR as gold standard are listed in Table 3.

**Table 3:** Results of variplex test and RT-PCR assay performed simultaneously in 109 patients (95% CI)

Sensitivity	17.0% (7.6%–30.8%)
Specificity	88.7% (78.1–95.3%)
PPV	53.3% (26.6–78.7%)
NPV	58.5% (47.9–68.6%)
Accuracy	57.8% (48.0–67.2%)

## Discussion

Here, we report on the performance evaluation of a new and faster in vitro diagnostic method in patients of a general hospital following a nosocomial SARS-Cov-2 outbreak. This outbreak was detected in March 2020 at a normal care unit of the department of trauma surgery and geriatrics. COVID-19 was confirmed by RT-PCR assays performed by an external institute, and latency of results was significant at that time. For efficient SARS-CoV-2 screening of every person in our hospital, i.e., all patients and the whole staff, as well as for real-time results, we decided to use a point-of-care device based on LAMP technology as recommended by others [6, 11].

The potential of LAMP technology has been demonstrated impressively. Synthesized RNA of SARS-CoV-2 could be amplified to detectable levels in dilutions as low as 2–100 copies per reaction [11, 12]. Sensitivity of LAMP in detecting intact viral RNA, which was extracted from cell culture supernatants of isolates from COVID-19 patients, has been reported to be tenfold lower than that of qRT-PCR (10^−7^ versus 10^−8^ dilutions), while specificity was high against all viruses tested [10]. In clinical specimens, sensitivity and specificity of LAMP after RNA extraction were 100 and 98.7–100%, respectively [10, 13]. In general, nucleic acid-based methods are thought to be sensitive but prone to false-positive [14].

After introduction of the variplex test system end of March 2020 in our hospital, we rarely observed discrepant results compared with RT-PCR suggestive of false-positive. However, negative variplex test results in patients with CT-scans typical for COVID-19 as well as with positive RT-PCR results were noticed. Therefore, since 15th of April, we increased sample volume (SLSolution) from 75 to 100 μL according to the manufacturers’ advice to increase sensitivity. When observations of false-negative variplex test results persisted, we performed an interim analysis. Endpoint of the interim analysis was the false-negative rate of variplex test system compared with RT-PCR in all patients admitted in April 2020.

The false-negative rate of the variplex SARS-CoV-2 test system compared with RT-PCR in all patients admitted to our general hospital in April 2020, in whom simultaneous swabs could be obtained (*n* = 109), was 83% and sensitivity was 17%. As a consequence, we discontinued variplex testing without RNA extraction by routine and initiated this study.

In view of these disappointing results, we analyzed the whole process from the technique of throat swabs to the release of variplex test results. SARS-CoV-2 viral load in upper respiratory specimens of infected patients decreases in the course of the disease [1]. This temporal dynamics in viral shedding [15] could be the reason for negative results, which depend on the type of clinical specimen. While positive rates, i.e., sensitivity in bronchoalveolar lavage fluid, are highest with 93%, positive rates with nasal swabs are 63% and with pharyngeal swabs 32%, respectively, as reported by Wang et al., however, the number of specimens analyzed were different and partly very low (BAL *n* = 15, nasal swabs *n* = 8, pharyngeal swabs *n* = 398) [16]. Diagnostic yield depends on sampling and therefore on swabs. In our hospital, simultaneous swabs were taken by well trained nurses, both oropharyngeal or nasopharyngeal, using standard swabs. Swabs with short fiber strands such as FLOQSwabs (Copan) may be superior compared with standard swabs with rayon flocking. However, identical swabs were used for variplex test as well as for RT-PCR assay. Unfortunately, due to pandemic caused scarcity of resources, only standard swabs were available in our hospital in April 2020.

## Conclusion

LAMP technology may be the answer to the urgently needed rapid, robust, and cost-efficient tests for the detection of COVID-19. Linked to obligate RNA extraction before isothermal amplification, excellent sensitivity and specificity up to 100% has been reported. However, without RNA extraction, the variplex test system failed to reliably detect SARS-CoV-2 directly in respiratory samples.
